# Long-COVID: Langzeitsymptome und morphologische/radiologische Korrelate

**DOI:** 10.1007/s00117-021-00910-7

**Published:** 2021-09-23

**Authors:** Majda M. Thurnher, Wolfgang Reith, Alexander P. Thurnher, Paulus Rommer

**Affiliations:** 1 Universitätsklinik für Radiologie und Nuklearmedizin, Allgemeines Krankenhaus Wien, Währinger Gürtel 18–20, 1090 Wien, Österreich; 2 Diagnostische Neuroradiologie, Universitätsklinikum des Saarlandes, Homburg/Saar, Deutschland; 3 Present Address: Medizinische Universität Innsbruck, Innsbruck, Österreich; 4 Allgemeines Krankenhaus Wien, Universitätsklinik für Neurologie, Wien, Österreich

**Keywords:** Coronavirus-Krankheit 2019, SARS-CoV‑2, Postakute Symptome, Spätfolgen, Long haulers, Coronavirus diseases 2019, SARS-CoV‑2, Post-acute symptoms, Delayed sequelae, Long haulers

## Abstract

**Hintergrund:**

Neurologische, pulmonale, kardiale und gastrointestinale Funktionsstörungen können in der postakuten Phase fortbestehen und ein *Long-COVID-Syndrom* bilden, das auch als *postakute Folgeerscheinungen der SARS-CoV-2-Infektion* (PASC) bezeichnet wird. Einige Patienten entwickeln trotz einer zu Beginn relativ milden Erkrankung anhaltende und schwächende Symptome und werden als „COVID-19 long haulers“ bezeichnet.

**Fragestellung:**

Vorstellung von Symptomen, Anzeichen und Biomarkern bei Patienten, die zuvor an COVID-19 erkrankt waren und Erörterung der möglichen zugrunde liegenden Mechanismen und Folgen.

**Methoden:**

Bestehende Literatur und berichtete Fälle sowie Expertenmeinungen werden analysiert und diskutiert.

**Ergebnisse:**

Das Long-COVID-Syndrom betrifft Überlebende von COVID-19 in allen Schweregraden der Erkrankung, selbst in leichten bis mittelschweren Fällen und bei jüngeren Erwachsenen, die keine Beatmungsunterstützung oder Krankenhaus- bzw. Intensivpflege benötigten. Problematisch ist, dass bei vielen Langzeitüberlebenden nie ein Labornachweis für COVID-19 erbracht wurde, was die Skepsis weckt, dass ihre anhaltenden Symptome eine physiologische Grundlage haben. Andererseits können einige Symptome, die bei einer postakuten COVID-19-Erkrankung auftreten, Folge einer kritischen Erkrankung oder eine Nebenwirkung von Behandlungen sein.

**Schlussfolgerung:**

Da es sich bei COVID-19 um eine neue Krankheit handelt, lässt sich nicht feststellen, wie lange diese Auswirkungen anhalten werden. Eine langfristige Überwachung der postakuten COVID-19-Symptome und ein Screening auf häufige Komorbiditäten sind unerlässlich.

## Abstract (en)

**Background:**

Neurologic, pulmonary, cardiac and gastrointestinal functional disorders can persist in the post-acute phase and constitute a long COVID syndrome, also known as post-acute sequelae of SARS-CoV‑2 infection (PASC). Some patients develop persistent and debilitating symptoms despite a relatively mild illness at onset and they are known as COVID-19 long haulers.

**Objective:**

Presentation of symptoms, signs and biomarkers present in patients previously affected by COVID-19 and discussion of possible underlying mechanisms and consequences.

**Methods:**

Existing literature and reported cases as well as expert opinions are analyzed and discussed.

**Results:**

Long COVID affects survivors of COVID-19 at all levels of disease severity, even in mild to moderate cases and younger adults who did not require respiratory support, hospitalization or intensive care. A challenging aspect is that many long haulers never had laboratory confirmation of COVID-19, raising skepticism that the persistent symptoms have a physiological basis. On the other hand, some symptoms seen in post-acute COVID-19 may occur as a consequence of critical illness or as a side effect of treatment.

**Conclusion:**

Given that COVID-19 is a new disease, it is not possible to determine how long these effects will last. Long-term monitoring of post-acute COVID-19 symptoms and screening for frequent comorbid conditions are essential.

Das schwere akute respiratorische Syndrom Coronavirus Typ 2 („severe acute respiratory syndrome coronavirus type 2“, SARS-CoV-2) hat weltweit zu über 117 Mio. bestätigten Infektionen und 2,6 Mio. Todesfällen durch die Coronavirus-Krankheit 2019 (COVID-19) geführt (Stand 11. Juli 2021; https://covid19.who.int).

## Definition

Neurologische, pulmonale, kardiale und gastrointestinale Dysfunktionen können in der postakuten Phase bestehen bleiben und ein sog. Long-COVID-Syndrom hervorrufen, das kürzlich auch als Syndrom der „post-acute sequelae of SARS-CoV‑2 infection“ (PASC) bezeichnet wird. Einige Patienten entwickeln trotz eines relativ leichten Krankheitsverlaufs anhaltende Symptome und sind als „COVID-19 long haulers“ bekannt [31].

„COVID long haulers“ bezeichnet im Allgemeinen die Personen mit COVID-19, bei denen > 28 Tage nach der Diagnose Symptome auftreten (Long-COVID), und dies unabhängig davon, ob labor- oder klinisch bestätigt. Die Symptome sind so heterogen wie bei der akuten COVID-19-Erkrankung und können konstant sein, schwanken oder durch Symptome anderer Organsysteme ersetzt werden. Ein weiteres Merkmal von Long-COVID ist, dass es Überlebende von COVID-19 jeden Schweregrads der Krankheit betrifft. Studien haben eindeutig gezeigt, dass auch Patienten nach einem leichten und mittelschweren COVID-19-Verlauf, jüngere Erwachsene sowie Patienten, die keine Atemunterstützung, Krankenhaus- oder Intensivpflege benötigten, Long-COVID widerfahren kann.

Eine kürzlich vorgeschlagene Einteilung der Symptomatik „population-based framework for symptomatic SARS-CoV‑2 infection“ unterteilt die COVID-19-Symptome basierend auf ihrem Beginn in 3 Phasen:

1. akute Infektion,
2. postakute hyperinflammatorische Erkrankung,
3. Spätfolgen (Tab. 1; [7]).

**Table 1:** 

	Akute COVID-19-Infektion	Postakute hyperinflammatorische Erkrankung	Spätfolgen
Charakteristika	Akute virale Replikation Initiale Immunantwort	Dysregulation der Immunantwort	Pathophysiologische Mechanismen noch unklar
Klinik	Fieber, Husten, Atemnot, Kopfschmerzen, Halskratzen, Erbrechen, Diarrhoe, Anosmie, Dysgeusie, Bauchschmerzen	Gastrointestinale, kardiovaskuläre dermatologische, pulmonale, neurologische, muskuloskeletale Manifestationen	Kardiovaskuläre, pulmonale, neurologische und psychiatrische Symptome
Laborparameter	Virusnachweis + Antikörpernachweis + (nach 2 Wochen)	Virusnachweis ± Antikörpernachweis + (nach 2 Wochen)	Virusnachweis und Antikörpernachweis unklar

## Frequenz

Um die tatsächliche Häufigkeit von Post-COVID-Symptomen zu beurteilen, ist es entscheidend, zwischen COVID-19-Patienten mit und ohne Krankenhausaufenthalt sowie Patienten, die eine intensivmedizinische Betreuung benötigten, zu unterscheiden. Beim Vergleich der Ergebnisse verschiedener Studien sind die Charakteristika der Kohorte (Alter der Population, Komorbiditäten, Fragebogentyp) zusätzlich zu berücksichtigen. Eine wichtige Einschränkung von publizierten Studien an COVID-19-Überlebenden ist die Tatsache, dass nicht alle Patienten persönlich untersucht wurden. Ein signifikanter Prozentsatz dieser Patienten wurde durch Televisiten gesehen [12] oder telefonische Screening-Tools (C19-YRS-Tool) kontaktiert [32]. Darüber hinaus wurde die Lebensqualität von COVID-19-Patienten oder kognitive Bewertungen nicht systematisch untersucht.

Der erste Bericht über physische und psychische Symptombelastung, Blutmarker und Bildgebung nach COVID-19-Erkrankung umfasste 384 Personen [24]. Anhaltende Symptome und radiologische Anomalien wurden bei einem signifikanten Anteil der Patienten gefunden (276/384). In die Studie eingeschlossen wurden jedoch nur SARS-CoV-2-positiv getestete Patienten, die einen längeren Intensiv- und stationären Aufenthalt hatten.

Eine systematische Analyse anhaltender Symptome bei Patienten mit COVID-19, basierend auf der Literaturrecherche mit 45 Studien und 9751 Teilnehmern, ergab einen hohen Prozentsatz (72,5 %) der Personen mit mindestens einem anhaltenden Symptom 60 Tage oder mehr nach der Diagnose [26]. Die am häufigsten berichteten Symptome waren Müdigkeit und Kurzatmigkeit sowie atypische Brustschmerzen. In einer Metaanalyse von 18.251 Publikationen wurden 15 Studien gefunden, in denen Langzeitwirkungen von COVID-19 erwähnt wurden [20]. Insgesamt wurden 55 Langzeiteffekte genannt, wobei Müdigkeit (58 %), Kopfschmerzen (44 %), Aufmerksamkeitsstörungen (27 %), Haarausfall (25 %) und Dyspnoe (24 %) am häufigsten auftraten. In die beiden Studien wurden hospitalisierte und nichthospitalisierte COVID-19-Patienten eingeschlossen.

Eine weitere Analyse von 31 Studien zu COVID-19-Folgen bei Erwachsenen unter 50 Jahren ergab folgende Symptome: anhaltende Müdigkeit (39–73 %), Atemnot (39–74 %), Beeinträchtigung der Lebensqualität (44–69 %), Beeinträchtigung der Lungenfunktion, Peri‑/Perimyo‑/Myokarditis (3–26 %), neurologische Symptome (55 %), erhöhte Inzidenz psychiatrischer Diagnosen und unvollständige Erholung der olfaktorischen und gustatorischen Dysfunktion (33–36 %; [38]).

Die Studie aus Großbritannien umfasste 100 *im Krankenhaus behandelte* COVID-19-Überlebende, die 4 bis 8 Wochen nach der Entlassung untersucht wurden [14]. Diese Studie befasste sich mit hospitalisierten Patienten mit und ohne Intensivaufenthalt. Die Ergebnisse zeigten, dass Patienten, die eine Intensivpflege benötigen, nach der Entlassung wesentlich häufiger über anhaltende Symptome wie Müdigkeit (72 % vs. 60,3 %) und Atemnot (65,6 % vs. 42,6 %) berichteten.

Im Mai 2021 wurde eine retrospektive Analyse dreier US-amerikanischer Patientendatenbanken zur Bewertung der Prävalenz persistierender Symptome nach der akuten Phase der SARS-CoV-2-Infektion bei 193.113 Erwachsenen zwischen 18 und 65 Jahren veröffentlicht [8]. Insgesamt 14 % der mit SARS-CoV‑2 infizierten Personen im Alter von ≤ 65 Jahren entwickelten mindestens eine neue Art von klinischen Folgeerscheinungen, die nach der akuten Phase der Erkrankung eine medizinische Versorgung erforderte.

In einer dänischen landesweiten Kohortenstudie, welche die Häufigkeit postakuter Auswirkungen einer SARS-CoV-2-Infektion bei 8983 *nichthospitalisierten Personen* untersuchte, wurden bei 1,2 % Atemnot, bei 0,2 % Husten, bei 0,4 % Kopfschmerzen, bei 0,2 % Müdigkeit und bei 0,3 % unspezifische Schmerzen festgestellt [22]. Eine französische Studie zeigte, dass der mittelfristige Verlauf von 150 Patienten mit leichtem oder mittelschwerem COVID-19-Verlauf ungünstig war: Zwei Drittel der Patienten berichteten noch an Tag 30 und Tag 60 über bestehende Symptome, mehr als ein Drittel fühlte sich noch krank oder in einem schlechteren klinischen Zustand an Tag 60 als zu Beginn von COVID-19. Diese anhaltenden Symptome waren signifikant häufiger bei Patienten zwischen 40 und 60 Jahren, denjenigen, die zur Beginn der Erkrankung hospitalisiert wurden und Patienten, die einen schweren COVID-19-Verlauf mit Dyspnoe erlitten hatten [3].

Eine norwegische prospektive Kohortenstudie mit 312 nichthospitalisierten COVID-19-Patienten zeigte einen hohen Prozentsatz an jungen (16 bis 30 Jahre) Patienten, die 6 Monate nach ihrer Ersterkrankung an anhaltenden Symptomen litten [2], welche unabhängig von der Schwere ihrer Erkrankung oder erhöhten Antikörpertitern waren. Als Symptome wurden Konzentrations- und Gedächtnisstörungen, Atemnot und Müdigkeit genannt [2].

**Figure 1:**
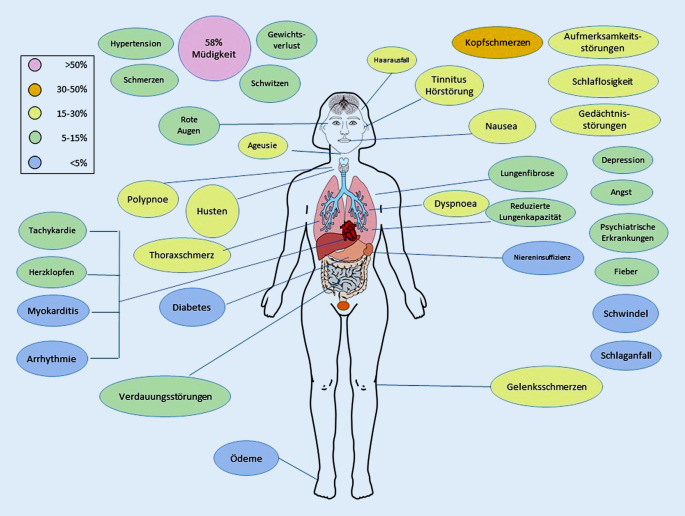

Interessanterweise wurde in einer Studie aus Wuhan berichtet, dass 3 bis 4 Wochen nach der Entlassung 86 % der 131 COVID-19-Patienten symptomfrei waren, nur 1,5 % an Kurzatmigkeit und 0 % an Fatigue litten (Abb. 1; [37]).

Long-COVID kann jeden treffen, auch junge Erwachsene, Kinder und Personen, die nur eine milde COVID-19-Erkrankung hatten und weder Atemunterstützung noch Krankenhausbehandlung benötigten!

## Neurologische Manifestationen

Die am häufigsten berichteten neurologischen Symptome im Zusammenhang mit Long-COVID waren: Müdigkeit, *Nebel im Gehirn* („brain fog“), Kopfschmerzen, Taubheitsgefühl/Kribbeln, Dysgeusie, Anosmie, Myalgien, Gedächtnis- und Konzentrationsstörungen [12]. Die häufigsten Komorbiditäten waren Depression/Angst (42 %) und Autoimmunerkrankungen (16 %).

*Fatigue* tritt in der Regel 100 Tage nach dem ersten COVID-19-Symptom auf und hat große Ähnlichkeit mit dem chronischen Erschöpfungssyndrom („chronic fatigue syndrome“, CFS). Dieses beinhaltet das Vorhandensein von schwerer handlungsunfähiger Müdigkeit, Schmerzen, neurokognitiver Behinderung, Schlafstörungen und Symptomen, die auf eine autonome Dysfunktion hindeuten. Eine geringfügige Zunahme der körperlichen und/oder der kognitiven Aktivität resultiert in einer Verschlechterung der globalen Symptome.

CFS wurde nach anderen Viruserkrankungen (Epstein-Barr-Virus, Zytomegalovirus, Enterovirus und Herpesvirus), aber auch nach immunologischen oder endokrin-metabolischen Dysfunktionen und neuropsychiatrischen Erkrankungen beschrieben. Bei weiblichen Patienten wurde häufiger über mäßige oder schwere Müdigkeit berichtet [14]. Graham et al. fanden Müdigkeit bei 85 % der Patienten [12]. Eine internationale Online-Umfrage unter 3762 Patienten, die nach eigenen Angaben Symptome im Zusammenhang mit COVID-19 aufwiesen, zeigte 7 Monate nach Ausbruch der Krankheit, dass 77,9 % weiterhin erschöpft waren [9]. Townsedet al. untersuchten 128 bestätigte COVID-19-Patienten und fanden heraus, dass durchschnittlich 10 Wochen nach den ersten COVID-19-Symptomen 52 % über anhaltende Müdigkeit berichteten und 31 % nicht zur Arbeit zurückgekehrt waren [34, 35]. Es ist bekannt, dass Atembeschwerden und Einschränkung der Lungenfunktion mit einer Fatigue einhergehen und diese bei COVID-19-Genesenen beachtet werden sollten [33].

Neben* Fatigue *wird häufig von der* myalgischen Enzephalomyelitis/dem chronischen Erschöpfungssyndrom *(„myalgic encephalomyelitis/chronic fatigue syndrome“, ME/CFS), umbenannt in *systemische Belastungsintoleranz*, von der CDC als eine behindernde und komplexe Krankheit definiert, berichtet. Schätzungsweise 836.000 bis 2,5 Mio. Amerikaner leiden an ME/CFS, und etwa 90 % der Menschen mit ME/CFS wurden nicht diagnostiziert (CDC) [11]. Die Pathologie von ME/CFS ist nicht bekannt: Eine multifaktorielle Genese und eine Fehlregulation mehrerer Kreisläufe als Reaktion auf einen bestimmten Auslöser wird diskutiert. Es gibt keine allgemein anerkannte Definition von ME/CFS. Bisher wurden 25 verschiedene diagnostische Kriterien vorgeschlagen [29]. Obwohl ME/CFS nicht ausschließlich als postinfektiöse Entität angesehen wird, wurde es mit mehreren Infektionserregern in Verbindung gebracht, darunter Epstein-Barr-Virus, Q‑Fieber, Influenza und andere Coronaviren. Es gibt unbestreitbare Ähnlichkeiten zwischen postakuten COVID-19-Symptomen und ME/CFS. Derzeit gibt es jedoch keine ausreichenden Beweise, um COVID-19 als infektiösen Auslöser für ME/CFS zu etablieren. Eine Beobachtungsstudie zur Untersuchung postakuter COVID-19-Symptome gemäß den ME/CFS-Kriterien wäre notwendig, um den Zusammenhang zu bestätigen.

Die Mechanismen hinter COVID-19-assoziierten *epileptischen Anfällen* sind noch nicht vollständig verstanden. Hypoxie, Multiorganversagen und Stoffwechselstörungen, die typischerweise bei schweren Erkrankungen auftreten, wurden als zugrunde liegende Ursache vorgeschlagen. Ein kürzlich veröffentlichter Fall zu epileptischen Anfällen mit Long-COVID bei einem 71-jährigen männlichen Patienten, der negativ auf SARS-CoV‑2 war, ohne einem anderen aufgedeckten provozierenden Faktor, führte dazu, dass epileptische Anfälle in die Liste der Symptome von Long-COVID aufgenommen wurde [19].

*Critical-Illness-Myopathie* (CIM) bei COVID-19 wurde bei Patienten veröffentlicht, die auf Intensivstationen behandelt wurden [39]. Ein vor kurzer Zeit veröffentlichter Fall zeigte jedoch eine Veränderung des Muskelmembranpotenzials und den Verlust von Myosin als Zeichen gleichzeitiger struktureller Veränderungen bei einem Patienten mit einem anscheinend milden Krankheitsverlauf von COVID-19 [28]. Dies deutet darauf hin, dass auch Myopathie Teil des Long-COVID-Spektrums sein könnte. Häufig ist die auftretende CIM als Folge des Intensivaufenthalts zu sehen.

*Neuropsychiatrische Symptome* bei COVID-19-Patienten können mit den direkten Auswirkungen der Infektion, zerebrovaskulären Erkrankungen (einschließlich Hyperkoagulation), physiologischen Beeinträchtigungen (Hypoxie), Nebenwirkungen von Medikamenten und sozialen Aspekten einer potenziell tödlichen Krankheit zusammenhängen. Erwachsene haben ein doppeltes Risiko, nach der COVID-19-Diagnose neu mit einer psychiatrischen Störung diagnostiziert zu werden [30].

Die häufigsten COVID-19-assoziierten psychiatrischen Erkrankungen waren Angststörungen, Schlaflosigkeit und Demenz. In einer Online-Umfrage bei 2291 Patienten berichteten 57,1 % der Teilnehmer von schlechter Schlafqualität, 32,1 % von Angst, 41,8 % von Stress und 7,6 % von einer posttraumatischen Belastungsstörung im Zusammenhang mit COVID-19 [1, 4]. Cortés-Alvarez et al. veröffentlichten eine Online-Umfrage mit 1105 Personen aus 32 Bundesstaaten in Mexiko; 15,7 % berichteten von mittelschweren bis schweren depressiven Symptomen, 22,6 % von mittelschweren bis schweren Angstsymptomen und 19,8 % von mittelschwerem bis schwerem Stress [6].

In einer Registerstudie mit 236.379 COVID-19-Überlebenden erhielt etwa ein Drittel innerhalb von 6 Monaten nach Auftreten der ersten Symptome eine neuropsychiatrische Diagnose. Überlebende auf der Intensivstation entwickelten eine um 56 % höhere Wahrscheinlichkeit, eine neuropsychiatrische Störung zu erleiden als Überlebende, die keine Intensivaufenthalt benötigten [34]. Inwieweit diese Symptome direkt auf COVID-19 zurückzuführen sind oder Folge von langen Intensivaufenthalten und der Pandemie mit allen ihren sozialen Aspekten zurückzuführen sind, bedarf weiterer Forschung.

In einer Studie wurden 29 COVID-19-Patienten eingeschlossen, wobei die häufigsten neurologischen Störungen den Geruchs- (25/29) und Geschmackssinn (29/29) betrafen [15]. 18/26 Patienten hatten im kognitiven MoCA-Test (Montreal Cognitive Assessment) auffällige Ergebnisse (mittlerer Score: 21,8/30 Punkte), wobei insbesondere frontoparietale Funktionen betroffen waren (z. B. Gedächtnis, Exekutivfunktionen und Visuokonstruktion; [15]). Die statistische Auswertung zeigte, dass eine hohe Korrelation der MoCA-Testwerte mit der Ausprägung der Stoffwechselerniedrigung im ^18^FDG-PET in diesen Hirnarealen nachweisbar war. Die postmortale Untersuchung einer verstorbenen Patientin, die das charakteristische Muster des zerebralen Glukose-Hypometabolismus zeigte, ergab eine deutliche Aktivierung von Mikroglia-Zellen vor allem in der weißen Substanz, wohingegen die kortikale graue Substanz relativ wenig betroffen war. Dies deutet darauf hin, dass die kortikalen Funktionen vor allem indirekt gestört waren.

## Neue Hypothesen

Es ist allgemein bekannt, dass SARS-CoV‑2 ein neurotropes Virus mit der Fähigkeit ist, neuronale Zellkulturen sowie Gehirnorganoide zu infizieren und zu replizieren. In mehreren Autopsiestudien wurden eindeutige Hinweise auf eine Beteiligung des Riechsystems und des Hirnstamms bei COVID-19 geliefert. Darüber hinaus weist der Hirnstamm eine relativ hohe Expression des ACE2-Rezeptors auf. Interessanterweise überschneiden sich Funktionen des Hirnstamms und Symptome von Long-COVID weitgehend. Die respiratorischen und kardiovaskulären Neuronenkreise des Gehirns sind im Hirnstamm eng miteinander verflochten. Es wird vermutet, dass eine Hirnstammdysfunktion an der Pathologie von Long-COVID beteiligt sein könnte [40].

## Geruchsstörungen

Eine postvirale olfaktorische Dysfunktion ist eine häufige Ursache, sowohl für kurz- als auch für langfristige Geruchsveränderungen. Der Prozentsatz der persistierenden Anosmie nach einer COVID-19-Erkrankung beträgt etwa 20 % (12–32 %; [20]). In einer Studie wurden Computertomographie (CT) und Magnetresonanztomographie (MRT) bei 23 Patienten mit persistierender COVID-19-Geruchsdysfunktion ausgewertet, und eine signifikante Anzahl von Patienten hatte radiologische Anomalien [18]. 91,3 % der Patienten hatten eine diffus erhöhte Signalintensität des Riechkolbens, hyperintense Foci oder Mikroblutungen. Eine offensichtliche Verklumpung der Riechhäute wurde in 34,8 % der Fälle und eine Ausdünnung mit Mangel an Riechhäuten in 17,4 % der Fälle festgestellt. Eine primäre olfaktorische kortikale Signalanomalie wurde in 21,7 % der Fälle gesehen [18]. Der Vergleich mit der Kontrollgruppe wurde jedoch nicht durchgeführt.

Eine weitere Studie zeigte bei Patienten mit anhaltender SARS-CoV-2-induzierter Riechstörung beidseitig geringere Riechkolbenhöhen [36]. In einer ^18^F‑FDG-Gehirn-PET-Studie bei 2 Patienten mit bestätigter SARS-CoV-2-Diagnose, die im postviralen Stadium der Erkrankung untersucht wurden, wurde ein Hypometabolismus des Gyrus olfactorius/rectus festgestellt [13].

## Radiologische Long-COVID-Studien

Lu et al. untersuchten mikrostrukturelle Veränderungen des Gehirns bei COVID-19-Patienten in einer MRT-basierten 3‑Monats-Follow-up-Studie [21]. Bei 60 COVID-19-Überlebenden und 39 Gesunden wurden eine Diffusions-Tensor-MRT-Bildgebung (DTI) und eine hochauflösende 3‑D-T1WI-Sequenz des Gehirns durchgeführt. In der Nachsorgephase wurden bei 55 % der COVID-19-Patienten neurologische Symptome festgestellt. COVID-19-Patienten hatten statistisch signifikant höhere bilaterale graue Substanzvolumina (GMV) in olfaktorischen Kortizes, Hippocampi, Insula, linkem Roland-Operculum, linkem Heschl-Gyrus und rechtem Gyrus cinguli und einer allgemeinen Abnahme der mittleren Diffusivität (MD), AD, RD begleitet von eine Zunahme von „fractional anisotropy“ (FA) in der weißen Substanz [21].

## Lungenmanifestationen

In einer Metaanalyse von 15 Studien, in denen Langzeit-COVID-Wirkungen betrachtet wurden, umfassten die Lungensymptome: Dyspnoe (24 %), Husten (19 %), Brustbeschwerden, verminderte Lungendiffusionskapazität (10 %), Schlafapnoe und Lungenfibrose [20]. Andere Studien berichteten über eine viel höhere Inzidenz (28 %, 53 %) von Lungenfunktionsstörungen bei COVID-19-Überlebenden [16]. Die Unterschiede in den Prozentsätzen sind hauptsächlich auf die Heterogenität der Nachbeobachtungszeiträume, die Definition von Lungenfunktionsstörungen und die Patientenpopulationen zurückzuführen. Neue oder verschlechterte Atemnot war Wochen nach der Entlassung bei hospitalisierten Patienten einer britischen Kohorte ein signifikantes Symptom und wurde bei zwei Fünftel der Stationspatienten und zwei Drittel der Intensivpatienten berichtet [14]. Es ist bemerkenswert, dass ein Fünftel der Patienten vor COVID-19 eine bereits bestehende Atemnot hatte und bei etwa 60 % eine bereits bestehende Atemwegserkrankung bestand [14].

## Dermatologische Manifestationen

Haarausfall nach COVID-19 wird als Telogen-Euvium angesehen und durch vorzeitige follikuläre Übergänge von der aktiven Wachstumsphase (Anagen) in die Ruhephase (Telogen) verursacht. Es ist ein bekanntes Phänomen nach systemischem Stress und Infektionen. Trotz der Tatsache, dass Post-COVID-Haarausfall ein selbstlimitierender Zustand ist, der ungefähr 3 Monate anhält, kann er emotionale Belastungen verursachen [25].

## Kardiovaskuläre Manifestationen

Herz-Kreislauf-Symptome bei COVID-19-Überlebenden umfassen Arrhythmien, Myokarditis und das posturale Tachykardiesyndrom (POTS; [5, 10]). Es wird angenommen, dass Entzündungen und ein erhöhter metabolischer und myokardialer Bedarf zu anhaltenden kardiovaskulären Symptomen beitragen, wie dies bei anderen schweren Coronavirus-Infektionen wie SARS (schweres akutes respiratorisches Syndrom; [23, 27]) beobachtet wurde. POTS ist durch eine Änderung der Herzfrequenz mit Positionsänderung gekennzeichnet, die oft von Herzklopfen und einer verminderten Belastungstoleranz begleitet wird [17]. POTS wurde zuvor mit postviralen Erkrankungen in Verbindung gebracht, aber der genaue Mechanismus ist unbekannt.

## Risikofaktoren

**Figure 2:**
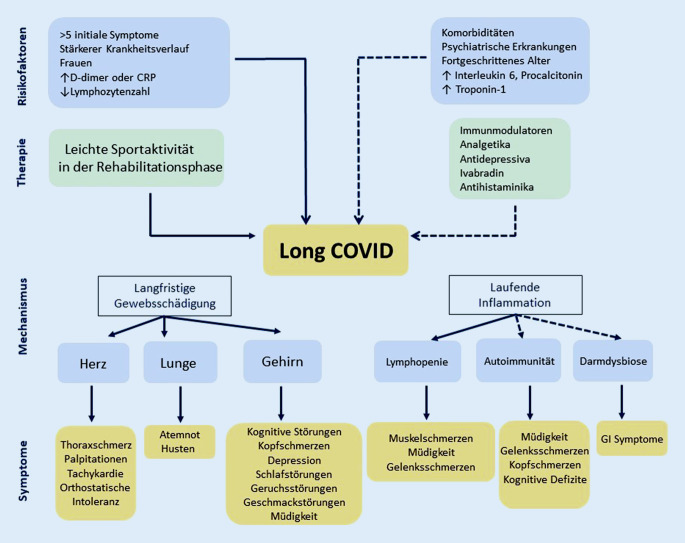

Über die Risikofaktoren für die Entwicklung von Long-COVID ist derzeit noch nicht viel bekannt. Das weibliche Geschlecht, mehr als 5 initiale Symptome, frühe Dyspnoe, bekannte psychiatrische Störungen und spezifische Biomarker (z. B. D‑Dimer, C‑reaktives Protein [CRP] und Lymphozytenzahl) sind als Risikofaktoren bereits etabliert ([41]; Abb. 2).

## Diskussion

Aus klinischer Sicht sollten sich Ärzte der Symptome, Anzeichen und Biomarker, die bei COVID-19-Patienten in verschiedenen Phasen auftreten können, bewusst sein. Das Ziel ist es, eine lange Progression von COVID-19 umgehend zu erkennen und zu stoppen und somit in weiterer Folge das Risiko chronischer Auswirkungen zu minimieren sowie zur Wiederherstellung der Gesundheit vor COVID-19 beizutragen.

Da es sich bei COVID-19 um eine neue Krankheit handelt, lässt sich nicht abschätzen, wie lange Long-COVID-Symptome anhalten werden. Viele „long haulers“ hatten nie eine Laborbestätigung von COVID-19, was die Skepsis aufkommen lässt, dass ihre anhaltenden Symptome eine physiologische Grundlage haben. Andererseits können einige Symptome, die bei postakutem COVID-19 beobachtet werden, als Folge einer kritischen Erkrankung oder als Nebenwirkung von medikamentösen Behandlungen wie Steroiden auftreten. Um ein besseres Verständnis zu erhalten, müssen zukünftige Studien nach Geschlecht, Alter, früheren Komorbiditäten, dem Schweregrad von COVID-19 und der Dauer jedes Symptoms stratifizieren.

Es ist schwierig zu unterscheiden, inwieweit viele der Symptome auf COVID-19 (Long-COVID) oder auf andere Ursachen zurückzuführen sind. Entsprechende Labortests existieren noch nicht, die eine einfache Zuordnung ermöglichen. Die soziale Isolation sowie Vorerkrankungen spielen ebenso eine Rolle und können möglicherweise durch die COVID-19 demaskiert werden. Zurzeit existieren nur wenige Empfehlungen oder Studien zur Behandlung von Long-COVID. Umso wichtiger erscheint es, die Patienten aufzuklären bzw. sie auf Neuigkeiten und veröffentlichte Empfehlungen hinzuweisen. Dies verlangt jedoch auch vom behandelnden Arzt eine entsprechende Vigilanz gegenüber neuen Entwicklungen. Eine umfangreiche Aufklärung des Patienten über die Symptome, Handlungsempfehlungen für ein angepasstes Rehabilitationsprogramm, Ergotherapie oder kognitive Therapie sind essenziell, ressourcensparendes Verhalten sowie das Einhalten ausreichender Pausen sind ebenfalls wichtig. Auf Komorbiditäten ist zu achten. Das Symptommanagement steht im Vordergrund. Therapeutika, z. B. Trizyklika, können Schmerzen, Depression und den Schlaf verbessern, sollten jedoch vor Anwendung auf Nebenwirkungen (z. B. kardiale Ereignisse) überprüft werden [42].

## Fazit für die Praxis

- Mit „COVID-19 long haulers“ werden im Allgemeinen Personen mit COVID-19 bezeichnet, bei denen > 28 Tage nach der Diagnose Symptome auftreten (Long-COVID).
- Der Schweregrad der initialen akuten Erkrankung korreliert nicht mit den Long-COVID-Symptomen.
- Insgesamt wurden 55 verschiedene Long-COVID-Symptome beschrieben; die häufigsten sind Müdigkeit, Konzentrations- und Gedächtnisstörungen, Angststörungen, Schlaflosigkeit, Kopfschmerzen, Atemnot, Haarausfall, Arrhythmien, Myokarditis und posturales Tachykardiesyndrom (POTS).
- Viele postakute COVID-19-Symptome ähneln einem postinfektiösen ME/CFS.
- Eine langfristige Überwachung der postakuten COVID-19-Symptome und ein Screening auf häufige Begleiterkrankungen sind unerlässlich.
- Die Erkenntnisse über Long-COVID sind noch unzureichend, entsprechende Therapieempfehlungen müssen im Verlauf je nach Erkenntnisse adaptiert werden.
